# Little Things That Kill

**Published:** 1888-02

**Authors:** 


### LITTLE THINGS THAT KILL.

At various times the newspapers have warned the public against swallowing the seeds of grapes, oranges &c., because of the danger of such substances getting into a small intestinal bag, or cul-de-sac, called by the doctors the appendix vermiformis. This is a little receptacle formed at the junction of the large and small intestines: but its use or object no physician knows. It has been thought to be a rudimentary or incomplete formation—or possibly some meaningless survival of a lost anterior type. At any rate, its existence, while presenting no apparent "reason for being," as the French say, is, on the other hand, a positive and constant source of danger, because of the liability of its becoming the receptacle of some undigested seed or other indigestible substance. In that case it produces a state of inflammation which in nearly all cases proves fatal.

Fortunately but few seeds, among the great number so heedlessly swallowed, seem to get into this little death-trap—although any one seems likely to lodge there. Perhaps more cases of inflammation of the bowels than the doctors suspect may be in reality due to this obscure and disregarded cause. One sad case, which to-day produces a feeling of deep regret among thousands, and which plunges a family into overwhelming grief, occurred in this city on Saturday evening, in the lamented death of J. Robert Dwyer, the much-esteemed adjutant of the Governor's Foot Guard—a man whose place that ancient corps cannot well make good. His case so baffled the physicians that an autopsy was had, and that revealed a piece of peanut shell in the *appendix vermiformis.*—*Hartford Times.*

